# Investigation into the antioxidant role of arginine in the treatment and the protection for intralipid-induced non-alcoholic steatohepatitis

**DOI:** 10.1186/s12944-015-0124-0

**Published:** 2015-10-14

**Authors:** Marwa M. Abu-Serie, Basiouny A. El-Gamal, Mohamed A. El-Kersh, Mohamed A. El-Saadani

**Affiliations:** Medical Biotechnology Department, Genetic Engineering and Biotechnology Research Institute, City for Scientific Research and Technology Applications (SRTA-City), New Borg El Arab, Alexandria, Egypt; Department of Clinical Biochemistry, College of Medicine, King Khalid University, Abha, Saudi Arabia; Biochemistry Department, Faculty of Science, Alexandria University, Alexandria, Egypt; College of Biotechnology, Misr University for Science and Technology, 6th of October City, Egypt

**Keywords:** Arginine (Arg), Nitric oxide (NO), Intralipid, Non-alcoholic steatohepatitis, Oxidative stress

## Abstract

**Background:**

This study investigated the possible roles of arginine (Arg) in ameliorating oxidative damage of intralipid (IL)-induced steatohepatitis (NASH).

**Methods:**

NASH was induced in Sprague-Dawley rats by intravenous administration of 20 % IL for three weeks and then rats were pre- and post-treated with intraperitoneal injection of Arg for two weeks. Several biochemical parameters (blood and hepatic lipid peroxidation, glutathione, glutathione peroxidase and superoxide dismutase, hepatic cytochrome P450 2El monooxygenase (CYP2E1), nitric oxide (NO), endothelial nitric oxide synthase (eNOS) and tumor necrosis factor-α “TNF-α”) and liver histopathology were detected for rat groups.

**Results:**

The administration of Arg either before or after IL significantly ameliorated uncontrolled elevation of TBARS content, CYP2E1 activity (0.32 ± 0.01 or 0.3 ± 0.02 IU/mg) and TNF-α level. These effects were associated with a significant increase in the levels of glutathione, activities of antioxidant enzymes, NO level (1.649 ± 0.047 or 1.957 ± 0.073 μmol/g) and activity of hepatic eNOS (0.05 ± 0.002 or 0.056 ± 0.002 IU/mg) compared to the IL-treated rats. Moreover, the injection of Arg in NASH-induced rats showed normal hepatocytes, no steatosis and no bile duct proliferation but mild inflammation in the group which received IL after Arg.

**Conclusions:**

These results proved that pre- and post-treatment with Arg blocked oxidative stress-induced NASH by inhibiting CYP2E1 activity, decreasing TNF- α level and restoration activities of eNOS and antioxidant enzymes as well as glutathione level. This antioxidant effect of Arg leads to reverse signs of liver pathology of NASH with amelioration of liver and kidney functions.

## Introduction

Arginine (Arg) is one of the most metabolically versatile amino acids, serves as a precursor for synthesis of nitric oxide (NO) and other biologically important compounds involved in cellular homoeostasis [1]. There has been considerable interest in the metabolism of Arg, primarily because it is the source of NO in biological tissues from its terminal guanidinium group by nitric oxide synthase (NOS). Some NOS isoforms (endothelial NOS (eNOS) and neuronal) are Ca dependent-constitutively expressed and Ca-independent inducible NOS (iNOS) isoform [2, 3]. Arg and its product NO possess potent antistress activity [4] thus Arg regulates cellular redox status and plays an effective role against oxidative stress [5, 6]. Oxidative stress plays a major role in the pathogenesis of several liver disorders, including non-alcoholic steatohepatitis (NASH) [7]. NASH is a severe phenotype of fatty liver (steatosis) with the development of necroinflammatory changes (hepatitis) that can progress to cirrhosis, with subsequent liver failure and an increased risk of hepatocellular carcinoma [8].

Free radicals accelerate the onset and progression of NASH [9]. An overload of free radicals cannot gradually be destroyed leads to generate deleterious processes that can seriously alter structure and functions of the cell membranes and macromolecules. Free radicals also enhance abnormal synthesis of several cytokines leading to hepatic necroinflammatory changes [10, 11] which mediated mainly through tumor necrosis factor-α (TNF-α). TNF-α has been incriminated to play an important pathogenic role in NASH, possibly partly related to its ability to induce oxidative stress [12, 13]. The body has several mechanisms to counteract oxidative stress by producing antioxidants, which are either naturally produced *in situ*, or externally supplied through foods. The endogenous cellular defense system consists of enzymatic scavengers (superoxide dismutase (SOD), glutathione peroxidase (GPx) and others) and non-enzymatic scavenger components such as reduced form of glutathione (GSH) [14]. Several previous studies have demonstrated the importance of GSH, SOD, and GPx in protecting liver against oxidative damage [15, 16] which may be mediated by hepatic microsomal cytochrome P450 2El monooxygenase (CYP2El). CYP2El induces fatty acid oxidation in NADPH-dependent manner producing prooxidant species, which if not encountered by antioxidants, may induce steatohepatitis [17].

Animal models of NASH may be divided into two broad categories: those caused by genetic mutation and those with an acquired phenotype produced by dietary or pharmacological manipulation [18]. Natural nutritional models have been described, including the use of fat-rich diet that aggravates oxidative stress, leading to steatohepatitis and provides a system for screening therapeutic targets to treat NASH [19]. In our previous study, El-Gamal et al (2006) [20], we reported that intralipid (IL) induced hyperglycemia and dyslipidemia and increased significantly body weight of rats which become normalized by the injection of Arg (500 mg/Kg) for 14 days either before or after the IL administration. In the present study, we investigated further possible roles of Arg administration in ameliorating oxidative damage of IL-induced steatohepatitis.

## Materials and methods

### Experimental groups

Fifty male Sprague-Dawely rats weighing from 110-134 were obtained from MISR University for Science and Technology (animal welfare assurance no. A5865-01). The animals were housed in cages with free access to standard chow (14 % protein, 4.5 % fat, 52.9 % carbohydrate, 9 % fiber) and water and kept under conventional conditions of temperature and humidity with 12 h light-12 h dark cycle. Rats were divided randomly into five groups; control group (the untreated normal rats), IL-treated group (rats received a daily dose of intravenous (i.v.) injection of 8 mL of 20 % IL/kg for three weeks), Arg-treated group (rats received a daily dose of intraperitoneal (i.p.) injection of 500 mg Arg/kg for two weeks), IL + Arg-treated group (rats i.v. injected with 20 % IL for three weeks then treated with 500 mg Arg/kg, i.p. daily for two weeks) and Arg + IL-treated group (rats received 500 mg Arg/kg daily for two weeks and injected with 8 mL of 20 % IL for three weeks).

At the end of the experimental period, all rats were sacrificed by decapitation under diethyl-ether anesthesia after an overnight fasting. Blood samples and liver tissues were collected.

### Determination of blood and hepatic lipid peroxidation product levels

Lipid peroxidation was determined by measuring thiobarbituric acid reactive substances (TBARS) in the plasma and liver tissues as described by Ohkawa et al. (1979) [21]. Briefly, 50 μL of plasma or liver homogenates were mixed with 8.1 % SDS, 20 % acetic acid containing 0.27 M HCl, pH 3.5 and 0.8 % thiobarbituric acid (Sigma-Aldrich, USA) and heated at 95 °C for 60 min. After cooling, 2.5 mL of n-butanol: pyridine mixture (15:1 V/V) was added, centrifuged and the absorbances of this organic layer were measured at 532 nm.

### Determination of hepatic CYP2E1 activity

Activity of CYP2El in liver homogenate was measured according to the method of Waxman, et al. (1989) [22]. Briefly, liver samples were mixed with 0.1 M potassium phosphate buffer, pH 7.4 containing 8 mM aniline and 1 mM NADPH (Sigma-Aldrich, USA) and incubated for 60 min at 37 °C. The reaction was terminated with 40 % TCA, centrifuged, 400 μL of supernatants were incubated with 10 % sodium carbonate and 2 % phenol for 60 min. The absorbances of samples were read at 630 nm. Protein contents (mg) of liver samples were determined using a kit obtained from Biodiagnostics, Egypt.

### Determination of hepatic NO level and eNOS activity

Nitrate plus nitrite measurement represents NO production as described by the method of Garner et al. (1956) [23] and its concentration was used to assess eNOS activity as described by Giordano et al. (2002) [24] and Chang et al. (1998) [25]. Briefly, 500 μL of liver samples were added to the reaction mixture containing 0.1 M Arg, 1.25 mM NADPH, 0.5 μM FAD, 0.5 μM FMN, 1 μM BH_4_ (Sigma-Aldrich, USA) and 10 μM CaCl_2_. The mixture was incubated at 37 °C for 60 min. Five hundred microliters of the mixture were collected for NO measurement after heat inactivation, incubated with 0.96 μM NADPH, 5 U/mL nitrate reductase and 0.1 M sodium phosphate buffer saline (pH 7.4) for 3 h. Finally, Griess reagent was added to the previous mixture and absorbances of sample were measured at 540 nm. The total protein contents (mg) of samples were determined as described above.

### Determination of blood and hepatic GSH levels

Content of GSH was determined in plasma and supernatant of liver homogenates by the method of Ellman (1959) [26]. The proteins in liver samples or plasma were precipitated using 5 % TCA. Supernatants were incubated with Ellman’s reagent and 0.2 M potassium phosphate buffer, pH 8 for 10 min. The absorbances of the developed yellow color of samples were measured against blank at 412 nm.

### Determination of blood and hepatic GPx activity

Total GPx activity was measured in plasma and supernatants of liver homogenates using the method described by Rotruck et al. (1973) [27]. Briefly, 50 μL of plasma or liver samples and 50 mM Tris-HCl buffer, pH 7.6 containing 0.127 mM EDTA and 1.63 mM GSH (Sigma-Aldrich, USA) were added to 0.026 M cumene hydroperoxide and incubated at 37 °C for 5 min (samples). For control, 50 μL plasma or liver samples and the previous buffer were added to 1.63 mM GSH and incubated at 37 °C for 5 min. One milliliter of 15 % TCA was added to control and sample and supernatants were incubated with 19.8 mg % DTNB for 5 min. The absorbances of samples and control were read at 412 nm.

### Determination of blood and hepatic SOD activity

A simple and rapid method for the SOD activity in plasma and liver homogenate supernatant was described by Marklund (1974) [28]. In quartz cuvette, 1 mL of 20 mM Tris-HCl buffer, pH 8.2 containing diethylene triaminopentaacetic acid and 20 mM pyrogallol (Sigma-Aldrich, USA) were mixed with 20 μL of plasma, liver samples or serial concentrations of SOD (20-200 ng/mL). The change in the absorbance of sample and standard was measured at 420 nm after 60 s and 120 s.

### Determination of blood and hepatic TNF-α level

Livers were homogenized in phosphate buffer saline containing 0.05 % sodium azide, 0.5 % Triton X-100 and protease inhibitor cocktail, pH 7.2 and centrifuged at 12,000 xg for 10 min. TNF-α concentrations in plasma and liver homogenate supernatant were measured using a commercial rat TNF-α ELIZA kit (RayBio, USA).

### Assessment of liver and kidney functions

Activities of blood alanine aminotransferase (ALT) and aspartate aminotransferase (AST) and albumin, urea and creatinine levels were determined in plasma samples using kits purchased from Biodiagnostics, Egypt.

### Histopathological study of liver samples

The formalin-fixed liver specimens were dehydrated in ascending grades of alcohol then cleaned by immersion in xylene followed by impregnation in melted paraffin and embedding to form solid paraffin blocks. Then, blocks were cut into 5 μm thick sections which stained with hematoxyline and eosin (H&E) for examination of any histopathological changes.

### Statistical analysis

Data were expressed as mean ± standard error of the mean (SEM) by the multiple comparisons one-way analysis of variance (ANOVA) with probability (*p*) values < 0.05 considered statistically significant. (Pairwise comparison applied and significance level adjusted for multiple comparisons).

## Results

**Fig. 1:**
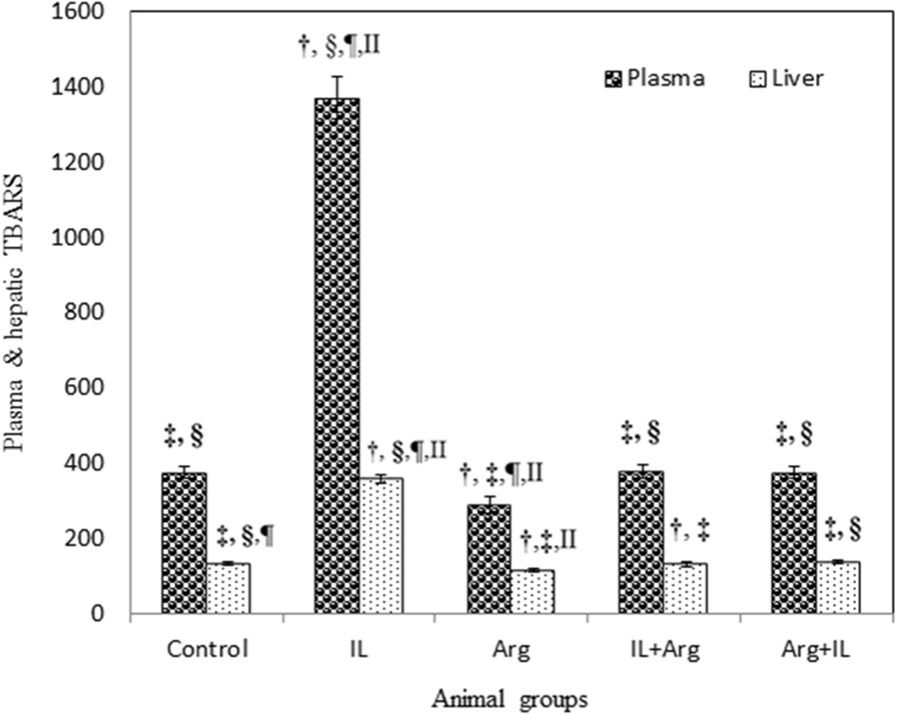
Levels of blood TBARS (nmol/L) and hepatic TBARS (nmol/g) in various animal groups. All values are expressed as the mean ± SEM and compared with: † control group; ‡ IL-treated group; § Arg-treated group; ¶ IL + Arg-treated group; II Arg + IL-treated group. Significance (*p* < 0.05)

**Fig. 2:**
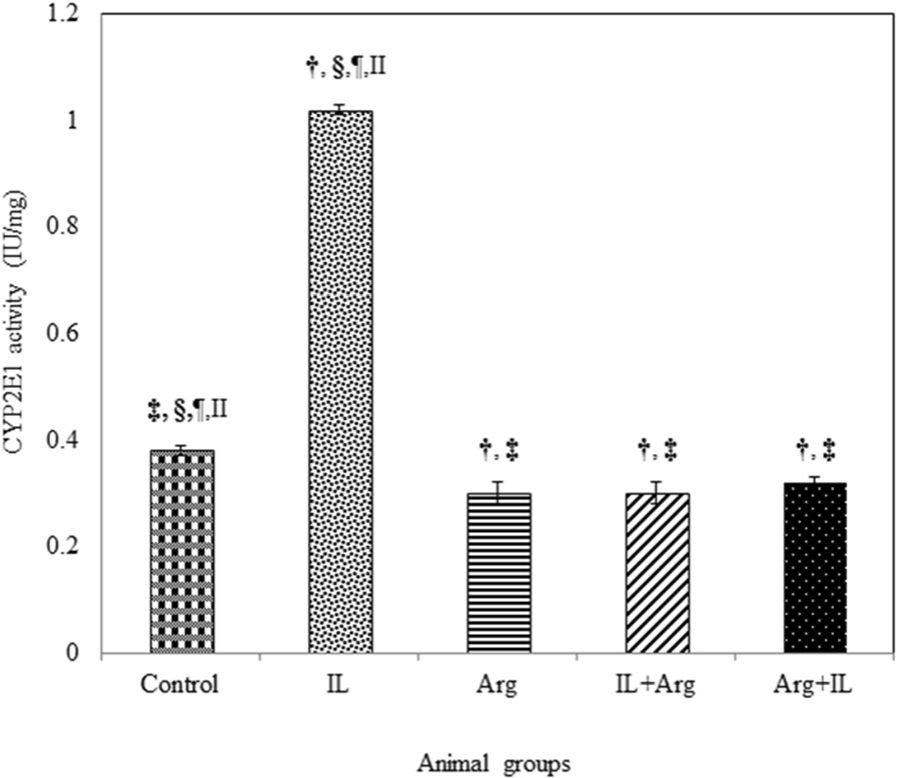
Hepatic activity of CYP2E1 (U/mg) in various animal groups. All values are expressed as the mean ± SEM and compared with: † control group; ‡ IL-treated group; § Arg-treated group; ¶ IL + Arg-treated group; II Arg + IL-treated group. Significance (*p* < 0.05)

**Fig. 3:**
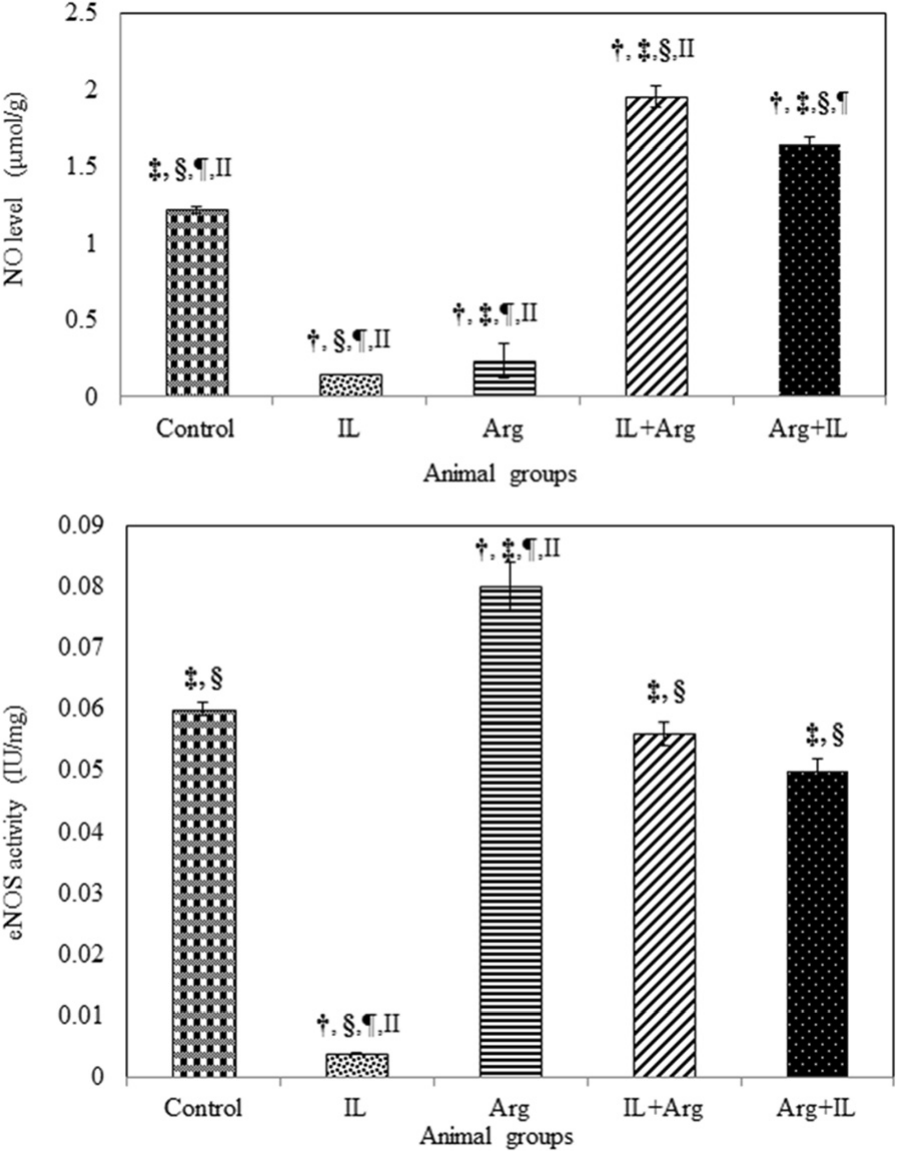
Hepatic level of NO (μmol/g) and hepatic activity of eNOS (U/mg) in various animal groups. All values are expressed as the mean ± SEM and compared with: † control group; ‡ IL-treated group; § Arg-treated group; ¶ IL + Arg-treated group; II Arg + IL-treated group. Significance (*p* < 0.05)

**Fig. 4:**
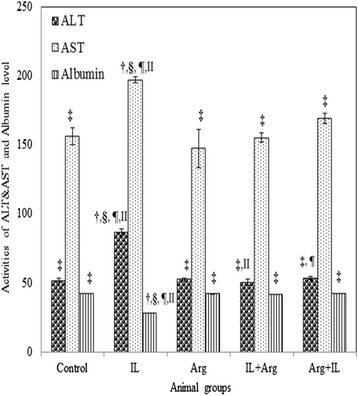
Activities (U/mL) of ALT and AST and blood level of albumin (mg/dl) in various animal groups0. All values are expressed as the mean ± SEM and compared with: † control group; ‡ IL-treated group; § Arg-treated group; ¶ IL + Arg-treated group; II Arg + IL-treated group. Significance (*p* < 0.05)

**Fig. 5:**
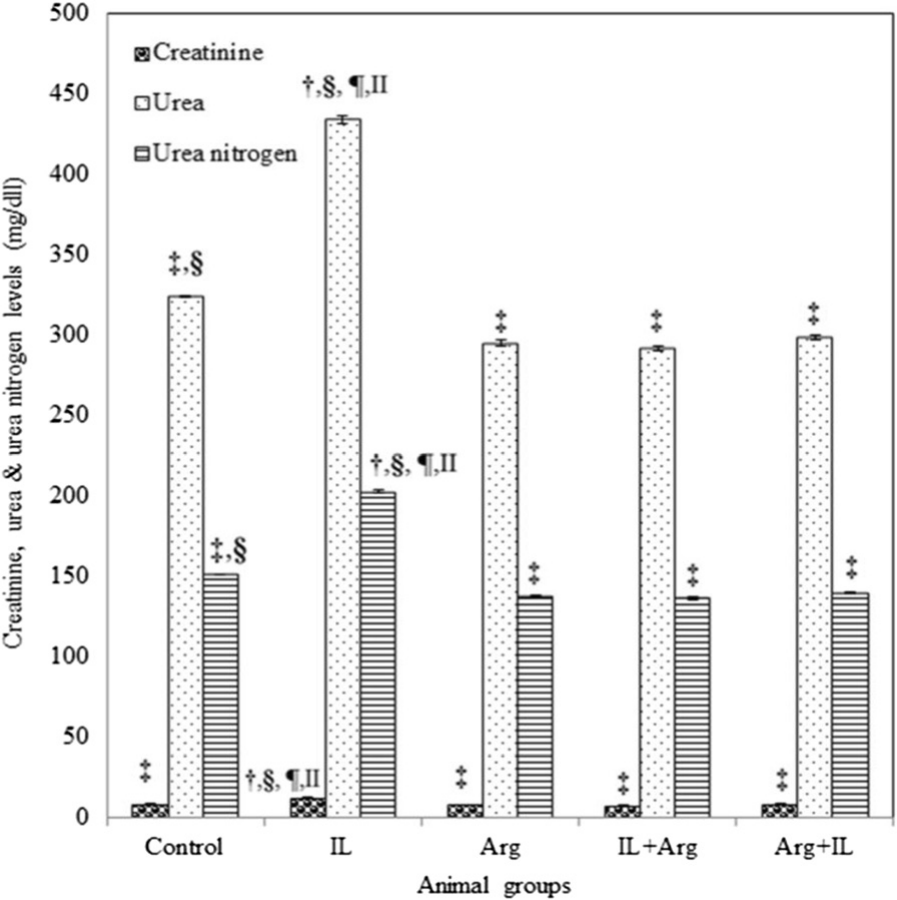
Blood level (mg/L) of creatinine, urea and urea nitrogen in various animal groups. All values are expressed as the mean ± SEM and compared with: † control group; ‡ IL-treated group; § Arg-treated group; ¶ IL + Arg-treated group; II Arg + IL-treated group. Significance (*p* < 0.05)

The intravenous administration of 20 % IL into rats produced a significant increase in the lipid peroxidation which was manifested by the elevation of blood and hepatic TBARS levels (1370 ± 55 nmol/L and 358.5 ± 10.6 nmol/g, respectively) as shown in Fig. 1. This significant elevation of TBARS may be attributed to a significant increase in the hepatic activity of CYP2E1 in IL group (1.02 ± 0.01 IU/mg) as shown in Fig. 2. In addition, Fig. 3 illustrates that IL administration resulted in a significant decrease in hepatic content of NO and hepatic activity of eNOS (0.147 ± 0.002 μmol/g and 0.004 ± 0.0001 IU/mg, respectively). Also, GSH content and activities of GPx and SOD decreased significantly below the control values in the blood and liver (Table 1). This oxidative stress associated with a mark elevation in blood and hepatic TNF-α levels (Table 2). Moreover, administration of IL resulted in a significant increase in activities of ALT and AST, as well as a significant decrease in blood albumin level and elevation in urea and creatinine levels, demonstrating liver and kidney dysfunction (Figs. 4 and 5).

**Table 1:** Levels of blood GSH (mg/L) and hepatic GSH (mg/g) and activities of blood & hepatic GPx and SOD (U/mg) in various animal groups

Group	Control	IL	Arg	IL + Arg	Arg + IL
Parameter					
Blood GSH	24.8 ± 0.7^‡,§,¶,II^	15.7 ± 0.8^†,§,¶,II^	41.6 ± 0.9^†,‡,¶,II^	34 ± 0.9^†, ‡, §^	33.4 ± 1.6^†, ‡, §^
Hepatic GSH	0.15 ± 0.001^‡,§,¶^	0.08 ± 0.01^†,§,¶,II^	0.2 ± 0.01^†,‡,¶,II^	0.19 ± 0.01^†,‡,§,II^	0.16 ± 0.01^‡,§, ¶^
Blood GPx	1.2 ± 0.01^‡,§,¶,II^	0.77 ± 0.03^†,§,¶,II^	1.3 ± 0.02^†,‡^	1.33 ± 0.02^†,‡,II^	1.27 ± 0.01^†, ‡,¶^
Hepatic GPx	3.6 ± 0.15^‡,§,¶,II^	2.9 ± 0.07^†,§,¶,II^	5.5 ± 0.19^†,‡,II^	5.44 ± 0.14^†,‡,II^	4.8 ± 0.14^†, ‡, §,¶^
Blood SOD	1.23 ± 0.04^‡,§^	1.1 ± 0.0^†,§,¶,II^	1.34 ± 0.0^†,‡^	1.33 ± 0.04^‡^	1.25 ± 0.04^‡^
Hepatic SOD	2.9 ± 0.07^§,¶,II^	2.6 ± 0.16^§,¶,II^	3.63 ± 0.09^†,‡^	3.62 ± 0.9^†,‡^	3.45 ± 0.1^†,‡^

All values are expressed as the mean ± SEM and compared with: † control group; ‡ IL-treated group; § Arg-treated group; ¶ IL + Arg-treated group; II Arg + IL-treated group. Significance (*p* < 0.05)

**Table 2:** Levels of blood and hepatic TNF-α (pg/mg) in various animal groups

Group	Control	IL	Arg	IL + Arg	Arg + IL
Parameter					
Plasma TNF-α	4.5 ± 0.08 ^‡^	14.8 ± 0.09^†,§,¶,II^	4.12 ± 0.13^‡^	4.43 ± 0.06^‡^	4.47 ± 0.09^‡^
Hepatic TNF-α	13.1 ± 0.6^‡^	111 ± 0.49^†,§,¶,II^	11.9 ± 0.5^‡^	13.02 ± 0.49^‡^	24.6 ± 0.6^‡^

All values are expressed as the mean ± SEM and compared with: † control group; ‡ IL-treated group; § Arg-treated group; ¶ IL + Arg-treated group; II Arg + IL-treated group. Significance (*p* < 0.05)

On the other hand, administration of Arg either before or after IL ameliorated significantly uncontrolled elevation of TBARS content in the blood (374 ± 16 or 376 ± 17 nmol/L) and liver (135.7 ± 3.5 or 129.2 ± 6.1 nmol/g) as well as the hepatic activity of CYP2E1 activity (0.32 ± 0.01 or 0.3 ± 0.02 IU/mg) and these values were close to the levels of control rat group (3.74 ± 0.15 nmol/mL, 132.1 ± 5.3 nmol/g and 0.38 ± 0.01 IU/mg, respectively). These effects were associated with a significant increase in the levels of GSH and activities of GPx and SOD in the blood and liver, compared to IL-treated rats (Figs. 1, 2 and Table 1). Also, hepatic NO level (1.649 ± 0.047 or 1.957 ± 0.073 μmol/g) and activity of hepatic eNOS (0.05 ± 0.002 or 0.056 ± 0.002 IU/mg) increased significantly compared to IL-treated rats (Fig. 3). In addition to both plasma and hepatic levels of TNF-α were ameliorated to be near to normal values (Table 2). Furthermore, pre- and post-treatment using Arg maintained the blood parameters of liver and kidney functions near to control values indicating the restoration of their normal functions (Figs. 4 and 5).

**Fig. 6:**
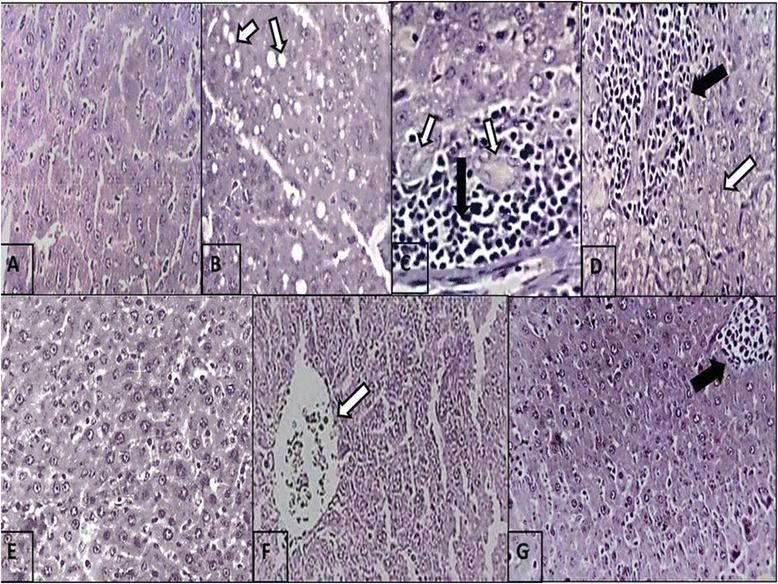
Photomicrograph of rat liver (H&E stain × 400). **a** rat control liver. (**b**, **c** and **d**) IL-treated rat liver showing **b** fatty vacuoles (*white arrows*), **c** inflammatory cell infiltration of portal tract (*black arrow*) and proliferation of bile duct (*white arrows*), and **d** necrosis (*white arrow*) with heavy inflammation (*black arrow*). **e** Arg-treated rat liver showing normal hepatocytes. **f** IL plus Arg treated rat liver showing normal hepatocytes with dilated blood vessel (*arrow*). **g** Arg plus IL treated rat liver showing normal hepatocytes with mild inflammation (*arrow*)

The histopathological study supported the results obtained from the biochemical tests. Figure 6a shows the normal architecture of hepatocytes of control rats, whereas IL-treated group demonstrates small fatty vacuoles in their cytoplasm and proliferation of bile ducts. Also, steatosis was detected with microvascular changes, including heavy inflammatory cells infiltration and clearing of cytoplasm and nuclei (necroinflammation) which are the histological hall markers of steatohepatitis (Fig. 6b, c, and d). In addition, histopathological examination of Arg group shows normal hepatocytes with dilating sinusoids (Fig. 6e) and IL + Arg and Arg + IL groups showed normal hepatocytes, no steatosis and no bile duct proliferation but mild inflammation in the group received IL after Arg (Fig. 6f and g).

## Discussion

Our previous study confirmed that intravenous administration of 20 % IL resulted in the elevation of hepatic lipid contents [20] which may deposit as lipid droplets in hepatocytes [29]. The hepatic steatosis has become the main cause of liver tests abnormalities [30] and increases the sensitivity of the liver to injury, necrosis and inflammation [10]. Hepatic steatosis leads to mitochondrial dysfunction that plays a key role in abnormal generation of free radicals [31]. In addition to mitochondrial dysfunction, the cumulative effect of extramitochondrial fatty acids oxidation represents a further increase in oxidative stress and mitochondrial impairment. The microsomal oxidation of fatty acids was catalyzed by CYP2E1 which enhanced its expression in IL-induced hyperlipidemia as it was proved in Ng et al. (2015) study on pigs [32].

Free radicals that initiate lipid peroxidation, depletion of antioxidants, destruction of membrane and oxidative damage of proteins lead to increased TBARS and decreased GSH level, GPx activity, SOD activity and albumin level after IL administration [33, 34]. This hepatic oxidative stress is tightly associated with TNF-α-mediated hepatic inflammation. TNF-α level was elevated significantly in nonalcoholic fatty liver disease and NASH in both humans and animals [13, 35]. Moreover, proinflammatory cytokines activate transcription of CYP2E1 [36] and iNOS. Over expression of iNOS enhances uncontrolled production of NO that favors the formation of ONOO- when it is accompanied by increasing O_2_^.-^ production; thus this reaction results in reduction of NO availability and eNOS inactivation [37, 38]. In the same time, hyperlipidemia which is developed after IL administration leads to eNOS deficiency and subsequently, decreases NO production [39, 40]. The resulting liver injury is associated with bile duct proliferation [41]. Also, hyperlipidemia that induces oxidative stress could exert their injurious activities in other extrahepatic tissues such as kidney. Scheuer et al. (2000) reported that high-fat diet induced hyperlipidemia led to a rise in glomerular tubulointerestitial generation of ROS leading to significant chronic tubulointerestitial damage was associated with elevation of serum level of urea and creatinine, indicating renal dysfunction [42].

On the other hand, this study provides evidence that daily administration of Arg whether before or after IL injection, leads to various beneficial effects. Arg injection resulted in a significant decrease in level of TBARS and CYP2El activity indicating that Arg reduced oxidative stress. This is consistent with previous results which proved that pre- and post-treatment with Arg lowered oxidative stress in animal model of hepatotoxicity [43]. Also, we found that this effect of Arg was associated with induction of GSH and activities of SOD, GPx and eNOS as well as NO level.

The Arg efficacy may be attributed to its direct antioxidant effect that is due to the alpha-amino group, a chemical moiety different from that necessary for NO generation. By acting as an antioxidant, Arg may scavenge O_2_^.-^ and thereby prevent eNOS-mediated O_2_^.-^ production in an uncoupled status. In addition to its indirect antioxidant effect by generation of NO [44], it is reported that NO prevents oxidative stress in tissues firstly by interrupting chain reaction of lipid peroxidation via forming non radical novel nitrogen-containing lipid adducts [45] and inhibiting the catalytic activity of CYP2E1 [46, 47]. Secondly, NO may trigger the expression of antioxidant enzymes and novel nitrosative stress resistance genes [48]. Lastly, NO augments the antioxidant potency of GSH [49] by forming S-nitrosoglutathione which is approximately 100 fold more potent than that of GSH [50]. GSH is not only a major antioxidant, but also upregulates GPx activity which protects against oxidation and nitration reactions [51].

Therefore, the ROS-scavenging property of Arg together with its ability to sustain spare endogenous antioxidants and decrease activity of the hepatic lipogenic enzyme may inhibit oxidative liver damage and decrease inflammation status [6, 47, 52]. Recent studies were reported that Arg inhibits uncontrolled synthesis of TNF-α thus block its deleterious effects [53, 54]. The experimental study of Ozsoy et al. (2011) [55] demonstrated that i.p. administration of 500 mg/kg Arg for 7 days significantly prevented the elevation of liver enzymes and decreased bile duct proliferation and leukocyte infiltration with no necrosis. Studies of Engin et al (2011) [56] and Nanji et al (2001) [47] proved that i.p. pre- and post-treatment of Arg provided a significant treatment and protection against the liver injury (necroinflammatory lesions, hepatocellular degeneration and fibrosis). Infusion of Arg in experimental animals increased renal plasma flow and glomerular filtration rate. So animals were treated with gentamicin or cisplatin-induced nephropathy together with Arg showed a significant amelioration in renal functions as indicated by blood urea nitrogen and creatinine levels [57, 58]. Moreover, this dose of Arg (500 mg/kg) has been clinically applied for several other human diseases [59–61].

## Conclusions

In this study, we have demonstrated that 2 weeks of pre- and post-treatment with Arg protected and treated liver against IL-induced NASH. This Arg effect is most likely attributed to its direct and NO dependent antioxidant property.

## Abbreviations

Arg: Arginine
CYP2E1: Cytochrome P450 2El monooxygenase
eNOS: Endothelial nitric oxide synthase
GPx: Glutathione peroxidase
GSH: Reduced form of glutathione
IL: Intralipid
NASH: Non-alcoholic steatohepatitis
NO: Nitric oxide
ROS: Reactive oxygen species
SOD: Superoxide dismutase
TBARS: Thiobarbituric acid reactive substances

## References

[1] Morris SM (2006). Arginine: beyond protein. Am J Clin Nutr.

[2] Boger RH, Bode-Boger SM (2001). The clinical pharmacology of L-arginine. Annu Rev Pharmacol Toxicol.

[3] Beaumier L, Castillo L, Yu YM, Ajami AM, Young VR (1996). Arginine: new and exciting developments for an “old” amino acid. Biomed Environ Sci.

[4] Gupta V, Gupta A, Saggu S, Divekar HM, Grover SK, Kumar R (2005). Anti-stress and adaptogenic activity of L-arginine supplementation. Evid Based Complement Alternat Med.

[5] Ranjbar K, Nazem F, Nazari A (2015). Effect of exercise training and L-arginine on oxidative stress and left ventricular function in the post-ischemic failing rat heart. Cardiovasc Toxicol.

[6] Suliburska J, Bogdanski P, Krejpcio Z, Pupek-Musialik D, Jablecka A (2014). The effects of L-arginine, alone and combined with vitamin C, on mineral status in relation to its antidiabetic, anti-Inflammatory, and antioxidant properties in male rats on a high-fat diet. Biol Trace Elem Res.

[7] Rolo AP, Teodoro JS, Palmeira CM (2012). Role of oxidative stress in the pathogenesis of nonalcoholic steatohepatitis. Free Radic Biol Med.

[8] Tetri LH, Basaranoglu M, Brunt EM, Yerian LM, Neuschwander-Tetri BA (2008). Severe NAFLD with hepatic necroinflammatory changes in mice fed trans fats and a high-fructose corn syrup equivalent. Am J Physiol Gastrointest Liver Physiol.

[9] Uetake Y, Ikeda H, Irie R, Tejima K, Matsui H, Ogura S (2015). High-salt in addition to high-fat diet may enhance inflammation and fibrosis in liver steatosis induced by oxidative stress and dyslipidemia in mice. Lipids Health Dis.

[10] Pessayre D, Mansouri A, Fromenty B (2002). Nonalcoholic steatosis and steatohepatitis: V. Mitohondrial dysfunction in steatohepatitis. Am J Physiol Gastrointest Liver Physiol.

[11] Tan X, Xie G, Sun X, Li Q, Zhong W, Qiao P (2013). High fat diet feeding exaggerates perfluorooctanoic acid-induced liver injury in mice via modulating multiple metabolic pathways. PLoS One.

[12] Diehl AM (2004). Tumor necrosis factor and its potential role in insulinresistance and nonalcoholic fatty liver disease. Clin Liver Dis.

[13] Haukeland JW, Damas JK, Konopsk Z, Loberg EM, Haaland T, Goverud I (2006). Systemic inflammation in nonalcoholic fatty liver disease is characterized by elevated levels of CCL_2_. J Hepatol.

[14] Pham-Huy LA, He H, Pham-Huy C (2008). Free Radicals, antioxidants in disease and health. Int J Biomed Sci.

[15] El-Boshy ME, Risha EF, Abdelhamid FM, Mubarak MS, Hadda TB (2015). Protective effects of selenium against cadmium induced hematological disturbances, immunosuppressive, oxidative stress and hepatorenal damage in rats. J Trace Elem Med Biol.

[16] Wang J, Zhu H, Yang Z, Liu Z (2013). Antioxidative effects of hesperetin against lead acetate-induced oxidative stress in rats. Indian J Pharmacol.

[17] Robertson G, Leclercq I, Farrell GC (2001). Nonalcoholic steatosis and steatohepatitis: II. Cytochrome P-450 enzymes and oxidative stress. Am J Physiol Gastrointest Liver Physiol.

[18] Anstee QM, Goldin RD (2006). Mouse models in non-alcoholic fatty liver disease and steatohepatitis research. Int J Exp Pathol.

[19] Matsuzawa N, Takamura T, Kurita S, Misu H, Ota T, Ando H (2007). Lipid-induced oxidative stress causes steatohepatitis in mice fed an atherogenic diet. Hepatology.

[20] El-Gamal B, El-Kersh M, Aboserie M, El-Saadani M (2006). Regulation of intralipid-induced dyslipidemia- hyperglycemia by exogenous L-arginine in experimental animals. J Med Res Inst.

[21] Ohkawa H, Ohishi N, Yagi K (1979). Assay for lipid peroxide in animal tissues by thiobarbituric acid reaction. Anal Biochem.

[22] Waxman DJ, Morrissey JJ, LeBlanc GA (1989). Female-predominant rat hepatic P-450 forms j (IIE1) and 3 (IIA1) are under hormonal regulatory controls distinct from those of the sex-specific P-450 forms. Endocrinology.

[23] Garner GB, Baumstark JS, Muhrer ME, Pfander WH (1956). Microbiological determination of nitrate. Anal Chem.

[24] Giordano A, Tonella C, Bulbarelli A, Cozzi V, Cinti S, Carruba MO (2002). Evidence for a functional nitric oxide synthase in brown adipocyte nucleus. FEBS Lett.

[25] Chang CI, Liao JC, Kuo L (1998). Arginase modulates nitric oxide production in activated macrophages. Am J Physiol.

[26] Ellman GL (1959). Tissue sulfhydryl groups. Arch Biochem Biophys.

[27] Rotruck J, Pope A, Ganther H, Swanson A (1973). Selenium: biochemical roles as a component of glutathione peroxidase. Science.

[28] Marklund S, Marklund G (1974). Involvement of the superoxide anion radical in the autoxidation of pyrogallol and a convenient assay for superoxide dismutase. Eur J Biochem.

[29] Cheng P, Yang Sh HXG, Zhou XY, Zhang YJ, Jin G (2011). Menin prevents liver steatosis through co-activation of peroxisome proliferator-activated receptor alpha. FEBS Lett.

[30] Bayard M, Holt J, Boroughs E (2006). Nonalcoholic fatty liver disease. Am Fam Physician.

[31] Paqout N, Delwaide J (2005). Fatty liver in the intensive care unit. Curr Opin Clin Nutr Metab Care.

[32] Ng K, Stoll B, Chacko S, Saenz de Pipaon M, Lauridsen C, Gray M (2015). Vitamin E in new-generation lipid emulsions protects against parenteral nutrition-associated liver disease in parenteral nutrition-fed preterm pigs. J Parenter Enteral Nutr.

[33] Valko M, Leibfritz D, Moncol J, Cronin MT, Mazur M, Telser J (2007). Free radicals and antioxidants in normal physiological functions and human disease. Int J Biochem Cell Biol.

[34] Browing JD, Horton JD (2004). Molecular mediators of hepatic steatosis and liver injury. J Clin Invest.

[35] Das SK, Balakrishnan V (2011). Role of cytokines in the pathogenesis of non-alcoholic fatty liver disease. Indian J Clin Biochem.

[36] Schattenberg JM, Czaja MJ (2014). Regulation of the effects of CYP2E1-induced oxidative stress by JNK. Redox Biol.

[37] Li JM, Shah AM (2004). Endothelial cell superoxide generation: regulation and relevance for cardiovascular pathophysiology. Am J Physiol Regul Integr Comp Physiol.

[38] Ceriello A (2003). New insights on oxidative stress and diabetic complications may lead to a “causal” antioxidant therapy. Diabetes Care.

[39] Musicki B, Liu T, Lagoda GA, Strong TD, Sezen SF, Johnson JM (2010). Hypercholesterolemia-induced erectile dysfunction: endothelial nitric oxide synthase (eNOS) uncoupling in the mouse penis by NAD(P)H oxidase. J Sex Med.

[40] Aktas C, Kanter M, Erboga M, Mete R, Oran M (2014). Melatonin attenuates oxidative stress, liver damage and hepatocyte apoptosis after bile-duct ligation in rats. Toxicol Ind Health.

[41] Kim F, Tysseling KA, Rice J, Pham M, Haji L, Gallis BM (2005). Free fatty acid impairment of nitric oxide production in endothelial cells is mediated by IkKB. Arterioscler Thromb Vasc Biol.

[42] Scheuer H, Gwinner W, Hohbach J, Grone EF, Brandes RP, Malle E (2000). Oxidative stress in hyperlipidemia-induced renal damage. Am J Physiol Renal Physiol.

[43] Saad EA (2012). Curative and protective effects of L-arginine on carbon tetrachloride-induced hepatotoxicity in mice. Biochem Biophys Res Commun.

[44] Lass A, Suessnbacher A, Wolkart G, Mayer B, Brunner F (2002). Functional and analytical evidence for scavenging of oxygen radicals by L-arginine. Mol Pharmacol.

[45] Rubbo H, Radi R, Trujillo M, Telleri R, Kalyanaraman B, Barnes S (1994). Nitric oxide regulation of superoxide and peroxynitrite-dependent lipid peroxidation. J Biol Chem.

[46] Wu D, Cederbaum A (2006). Nitric oxide donors prevent while the nitric oxide synthase inhibitor L-NAME increases arachidonic acid plus CYP2E1-dependent toxicity. Toxicol Appl Pharmacol.

[47] Nanji AA, Jokelainen K, Lau GK, Rahemtulla A, Tipoe GL, Polavarapu R (2001). Arginine reverses ethanol-induced inflammatory and fibrotic changes in liver despite continued ethanol administration. J Pharmacol Exp Ther.

[48] Klatt P, Lamas S (2000). Regulation of protein function by S-glutathiolation in response to oxidative and nitrosative stress. Eur J Biochem.

[49] Lube CB, Hayn M, Kitzmuller E, Vierhapper H, Lubec G (1997). L-arginine reduces lipid peroxidation in patients with diabetes mellitus. Free Radic Biol Med.

[50] Matsubara A, Tamai K, Matsuda Y, Niwa Y, Morita H, Tomida K (2005). Protective effect of polyethylene glycol-superoxide dismutase on leukocyte dynamics in rat retinal microcirculation under lipid hydroperoxide-induced oxidative stress. Exp Eye Res.

[51] Briviba K, Kissner R, Koppenol WH, Sies H (1998). Kinetic study of the reaction of glutathione peroxidase with peroxynitrite. Chem Res Toxicol.

[52] Wu LY, Fang YJ, Guo XY (2011). Dietary L-arginine supplementation beneficially regulates body fat deposition of meat-type ducks. Br Poult Sci.

[53] Quirino IEP, Carneiro MBH, Cardoso VN, dos Santos RGC, Vieira LQ, Fiuza JA (2014). Arginine supplementation induces arginase activity and inhibits TNF-α synthesis in mice spleen macrophages after intestinal obstruction. J Parenter Enteral Nutr.

[54] Bazarova SA, Alyavi AL, Dzhambekova GS, Kasymova GM (2013). The effect of L-arginine on the clinical and immunological parameters in patients with asthma. Inter J BioMed.

[55] Ozsoy Y, Ozsoy M, Coskun T, NamLi K, Var A, Ozyurt B (2011). The effects of L-arginine on liver damage in experimental acute cholestasis an immunohistochemical study. HPB Surg.

[56] Engin AB, Bukan N, Kurukahvecioglu O, Memis L, Engin A (2011). Effect of butylated hydroxytoluene (E321) pretreatment versus l-arginine on liver injury after sub-lethal dose of endotoxin administration. Environ Toxicol Pharmacol.

[57] Secilmis MA, Karatas Y, Yorulmaz O, Buvukafsar K, Sinqirik E, Doran F (2005). Protective effect of L-arginine intake on the impaired renal vascular responses in the gentamicin-treated rats. Nephron Physiol.

[58] Mansour MA, Al-Shabanah OA, El-Khashef HA (2003). L-arginine ameliorates kidney function and urinary bladder sensitivity in experimentally-induced renal dysfunction in rats. J Biochem Mol Biol.

[59] Parikh S, Saneto R, Falk MJ, Anselm I, Cohen BH, Haas R (2009). A modern approach to the treatment of mitochondrial disease. Curr Treat Options Neurol.

[60] Savoye G, Jemaa Y, Mosni G, Savoye-Collet C, Morcamp P, Dechelotte P (2006). Effects of intragastric L-arginine administration on proximal stomach tone under basal conditions and after an intragastric diet. Dig Dis Sci.

[61] Delles C, Schneider MP, Oehmer S, Fleischmann EH, Schmieder RE (2003). L-arginine-induced vasodilation of the renal vasculature is not altered in hypertensive patients with type 2 diabetes. Diabetes Care.

